# Unravelling the Complex of Substance Use and Suicide: Insights From a Qualitative Study in Pakistan

**DOI:** 10.1192/bjo.2024.208

**Published:** 2024-08-01

**Authors:** Muqaddas Asif, Salman Shahzad, Imran B. Chaudhry, Rakhshi Memon, Nusrat Husain

**Affiliations:** 1 Pakistan Institute of Living and Learning, Karachi, Pakistan; 2 University of Manchester, Manchester, United Kingdom; 3 University of Karachi, Karachi, Pakistan; 4 Ziauddin University, Karachi, Pakistan; 5 University College London, London, United Kingdom

## Abstract

**Aims:**

Suicide and substance use all contribute significantly to the global burden of mortality and morbidity. While existing evidence establishes the association between substance use and suicidal behaviour in Lower- and Middle-Income Countries (LMICs), only a few studies illustrate how substance use affected deceased people's lifestyles and suicide attempts. The study addresses this gap by exploring the role of substance use (particularly, alcohol and drug use) in overall lifestyles and suicides of deceased with substance use in Pakistan – an underexplored and under-researched country regarding suicide and substance use.

**Methods:**

We conducted in-depth qualitative interviews (N = 11) with close relatives and friends of those who died by suicide and have a history of substance use. The topic guide was comprised of a narrative part exploring the circumstances that surrounded the suicidal death of the deceased and a problem-focused part collecting comprehensive details about the deceased's personal, family, psychological, and social context and the role of substance use in the lifestyles and the suicide of the deceased.

**Results:**

The content analysis of interviews revealed five key themes: 1) Reasons for suicide, 2) Personality traits, 3) Psychological distress, 4) Initiation of substance use, and 5) Suicidal tendencies. Most of the participants reported the reason for their loved one's suicide was either an overdose of drugs or alcohol, family dynamics, or societal attitudes such as difficulty in building trust and finding acceptance within the family or society. Deceased individuals were perceived as impulsive with low control over their emotional states. Participants highlighted the underlying psychological distress in the deceased, emphasising the complexity of mental health and substance use problems. Participants reported that the deceased initiated drugs at an early age; had suicidal ideations; and overdosed themselves as a means of suicide.

**Conclusion:**

This study provides valuable insights into the role of substance use in suicide. The findings highlight the need for a holistic approach to understanding the multifaceted factors that may influence suicidal behaviours in individuals with substance use. Understanding these factors can help develop targeted suicide prevention and intervention strategies, particularly in low-resource settings such as Pakistan.

